# Investigation of Hepatitis C, D, and HIV Seroprevalence and Evaluation of APRI and FIB-4 Scores in HbsAg-Positive Patients

**DOI:** 10.3390/v17040568

**Published:** 2025-04-15

**Authors:** Fatih Mehmet Akıllı, Elif Nur Özbay Haliloğlu, Mehmet Mücahit Güncü, Dilara Turan Gökçe

**Affiliations:** 1 Department of Microbiology, Sincan Training and Research Hospital, Ankara 06930, Türkiye; 2 Department of Infectious Diseases and Clinical Microbiology, Sincan Training and Research Hospital, Ankara 06930, Türkiye; elifnurhaliloglu@gmail.com; 3 Department of Microbiology, Marmara University Institute of Health Sciences, Istanbul 34854, Türkiye; mguncu@marun.edu.tr; 4 Department of Gastroenterology, Sincan Training and Research Hospital, Ankara 06930, Türkiye; dilaraturan89@yahoo.com

**Keywords:** elimination, viral hepatitis, noninvasive scores, coinfection, vaccination, liver fibrosis, seroprevalence, epidemiology

## Abstract

This study aimed to assess the prevalence of HDV (hepatitis delta virus), HCV (hepatitis C virus), and HIV (human immunodeficiency virus) coinfections among HBsAg-positive patients and to determine the severity of liver fibrosis and biochemical markers. Furthermore, the study sought to evaluate the noninvasive fibrosis scores (APRI and FIB4) in predicting the severity of liver disease in patients with hepatitis B. A retrospective analysis of 1434 patients with chronic HBV admitted between January 2020 and December 2024 was conducted at Sincan Tertiary Hospital. The positivity rates of the following antibodies were the focus of the study: anti-HDV, anti-HCV, and anti-HIV. In addition to these, the levels of HIV-RNA, HCV-RNA and HBV-DNA, as well as several biochemical markers (ALT, AST, INR, albumin, bilirubin and platelet count) were also evaluated. The APRI and FIB-4 scores were calculated. Of the 1434 patients, 49 (3.4%) tested positive for anti-HDV, 784 were screened for anti-HCV, and 749 were screened for anti-HIV. The positivity rates were 3.4% (27/784) and 3.4% (26/749), respectively. According to ROC analysis, the FIB-4 score had a statistically significant effect on predicting anti-HDV negativity (AUC = 0.59, *p* = 0.031). However, the APRI score was not a significant predictor for anti-HDV positivity (AUC = 0.53, *p* > 0.05). APRI and FIB-4 scores did not have a statistically significant discriminatory power in predicting anti-HCV and anti-HIV positivity (*p* > 0.05). The cut-off value for the FIB-4 score in predicting anti-HDV positivity was 1.72, with a sensitivity of 61.4% and a specificity of 42.9% (*p* = 0.031). Among the HCV/RNA-positive patients (n = 5), all were male, and two also had positive anti-HBe results with undetectable HBV/DNA levels. One HIV/RNA-positive patient, a foreign national, was confirmed to have HIV/HBV/HDV infection. All HBsAg-positive patients should undergo routine anti-HDV testing. Vaccination programmes are vital in preventing the spread of HDV. Dual screening strategies are essential for identifying infected individuals and developing prevention and treatment programmes. Anti-HDV positivity indicates advanced liver fibrosis, emphasising the importance of screening and monitoring. However, the limited accuracy of the APRI and FIB-4 scores for detecting coinfections highlights the need to integrate noninvasive methods with molecular diagnostics for precise management.

## 1. Introduction

Hepatitis B (HBV) is considered to be a major global public health problem, with an estimated 258 million people with chronic infections and approximately 1.2 million new cases reported each year until 2022 [1]. HBV infection has been demonstrated to have a considerable impact on liver-related morbidity and mortality, with an estimated 1.1 million deaths per year attributable to this condition [2]. Türkiye is classified as a middle-to-high endemic region for HBV, with variability in prevalence across geographic and demographic groups [3]. The TURHEP study, a nationwide survey of 5460 individuals, reported an HBV surface antigen (HBsAg) positivity rate of 2.8%, reflecting the substantial burden of HBV in the country [3,4].

Coinfections with the hepatitis delta virus (HDV), hepatitis C virus (HCV), and human immunodeficiency virus (HIV) further complicate the clinical management of HBV. HDV, a satellite RNA virus that requires HBV replication, affects approximately 48–60 million individuals globally, with prevalence rates ranging from 0.8% in the general population to 13.02% among HBV carriers [2,5]. Türkiye has one of the highest HDV burdens worldwide, with the seroprevalence among HBsAg-positive individuals ranging from 2.84% to 46% [6,7]. HIV coinfection is observed in 5–15% of HBsAg-positive individuals, with 3–6 million people living with concurrent HBV and HIV infections globally [8,9,10]. Similarly, HCV coinfection rates among HBsAg-positive individuals range from 0.8% to 15%, significantly impacting disease outcomes [11]. These coinfections are associated with accelerated liver disease progression, including an increased risk of cirrhosis and mortality, compared with HBV monoinfection [7,12]. These findings underscore the importance of systematic screening and tailored management strategies for patients in high-risk groups, such as intravenous drug users and migrants from endemic regions [13].

The extent of liver fibrosis is not known with certainty, as the status of fibrosis is important in guiding the prognosis and treatment of HBV and HDV coinfection. The gold standard method of fibrosis assessment is biopsy, but this is an invasive procedure that is not performed at all centres [14]. Consequently, noninvasive methods and markers have recently been developed for the evaluation of liver fibrosis. In particular, the aspartate aminotransferase-to-platelet ratio index (APRI) and fibrosis-4 score (FIB-4) have been the subject of numerous studies and are also recommended by clinical guidelines. These scores are utilised to assess hepatic fibrosis in patients with chronic liver disease [14,15]. It is recommended by international guidelines and studies that HBsAg-positive individuals, especially those from high-risk populations, be routinely screened for HDV as a double reflex test [15,16]. Individuals afflicted with HDV coinfection have been observed to exhibit elevated FIB4 scores. However, this observation does not hold true when the APRI score is considered [17,18].

The objective of this study was to determine the prevalence of coinfections with HDV, HCV, and HIV among HBsAg-positive patients and to evaluate the severity of hepatic fibrosis and biochemical markers. In addition, the study sought to ascertain the value of noninvasive fibrosis scores (APRI and FIB4) in predicting the severity of liver disease in individuals.

## 2. Materials and Methods

### 2.1. Study Design and Population

The study was conducted at Sincan Training and Research Hospital from January 2020 to 2024. The study included HBsAg-positive samples, accompanied by confirmed data on HDVAg/Ab, anti-HBe, HBeAg, anti-HIV, anti-HCV, HBV DNA, HDV-RNA, HIV RNA, and HCV RNA levels, as well as various biochemical markers. To ensure data accuracy and prevent duplication, only the first sample from each patient was included in the analysis. All results were derived from the initial presentation of patients to our hospital, reflecting their baseline clinical and laboratory status. Patients with acute HBV infection, defined by anti-HBc IgM positivity or serum aspartate aminotransferase (AST) and alanine aminotransferase (ALT) levels exceeding 10 times the upper limit of normal, were excluded. Furthermore, individuals with a platelet count ≤150 × 10^9^/L, including those diagnosed with idiopathic thrombocytopenic purpura (ITP) or thrombotic thrombocytopenic purpura (TTP), were excluded from the study. Owing to the unavailability of a comprehensive treatment history, the analysis did not consider patients’ treatment status. The aforementioned exclusion criteria ensured a homogenous cohort for evaluating the study parameters and their clinical implications. This study was conducted with the approval of the Sincan Training and Research Hospital’s Clinical Research Ethics Committee (approval no: BAEK-2025-03). The study was carried out in accordance with the Helsinki Declaration.

### 2.2. Data Collection

The serological and molecular markers analysed included anti-HBs, HBeAg, anti-HBe, HBV-DNA, anti-HDV, HDV-Ag, anti-HIV, and anti-HCV. Biochemical parameters such as ALT and AST, serum albumin, total bilirubin, platelet count, and international normalised ratio (INR) values were retrieved from hospital records. Noninvasive fibrosis scores, including the FIB-4 score and the APRI score, were calculated via previously described formulae [19,20].

### 2.3. Laboratory Methods

Serological markers (HBsAg, anti-HBe, HBeAg, anti-HCV, and anti-HIV) were analysed via the ARCHITECT (Abbott, Abbott Park, IL, USA) and MaglumiX3-8 (Snibe, Shenzhen, Guangdong, China) systems. Anti-HDV seropositivity was assessed via the QIAsymphony^®^ DSP Virus/Pathogen Midi Kit (QIAGEN, Hilden, North Rhine-Westphalia, Germany). The quantification of HBV DNA was conducted via the Artus^®^ HBV QS-RGQ kit, whereas the assessment of HCV RNA and HDV RNA levels was performed via the Artus^®^ HCV QS-RGQ kit and real-time polymerase chain reaction (PCR) on the Rotor Gene Q platform (QIAGEN, Hilden, North Rhine-Westphalia, Germany).

### 2.4. Biochemical and Haematological Analysis

AST, ALT, albumin, and total bilirubin levels were analysed via Architect C16000 (Abbott, Abbott Park, IL, USA) and Beckman AU 5800 clinical chemistry analysers (Beckman Coulter, Brea, CA, USA). INR levels were measured via Sysmex CS-2500 coagulation analysers (Sysmex Corporation, Kobe, Hyogo, Japan). Finally, platelet counts were obtained from whole blood samples via Mindray BC 6000 (Mindray, Shenzhen, Guangdong, China), Siemens Advia 2120 (Siemens Healthineers, Erlangen, Bavaria, Germany), and ABX Pentra XL 80 haematology analysers (Horiba Medical, Montpellier, Occitanie, France).

### 2.5. Statistical Analysis

Data were analysed with the Statistical Package for the Social Sciences (SPSS) (IBM Corp., Armonk, NY, USA) 26.0 Statistics package programme. Descriptive characteristics of the patients were given as numbers and percentages. All data were presented as mean, standard deviation, median, minimum, and maximum. The suitability of the data for normal distribution was decided by looking at the skewness and kurtosis values. In the data set, non-normally distributed variables (z) and normally distributed variables (t) were presented as two groups, and the methods to be used in the analysis were selected accordingly. A Chi-Square Test was used to compare liver functions, fibrosis, and demographic parameters in HDV-positive and -negative patients. For normally distributed data, the comparison of various numerical parameters in HDV-positive and -negative patients was examined using an independent sample *t*-test. In addition, for non-normally distributed data, the Mann–Whitney U Test was used to compare various numerical parameters in HDV-positive and -negative patients. The relationships between HDV positivity and all other variables were analysed using a Spearman Correlation Analysis.

ROC analysis was performed to estimate the probability of HDV/Ab, HCV/Ab, and HIV/Ab positivity. The Logistic Regression Method was used to examine the effects of various variables on the probability of being HDV/Ab-, HCV/Ab-, and HIV/Ab-positive. In the whole study, significance levels were determined by considering 0.05 and 0.01 values. All the statistical results were evaluated at the 95% confidence level.

## 3. Results

**Figure 1:**
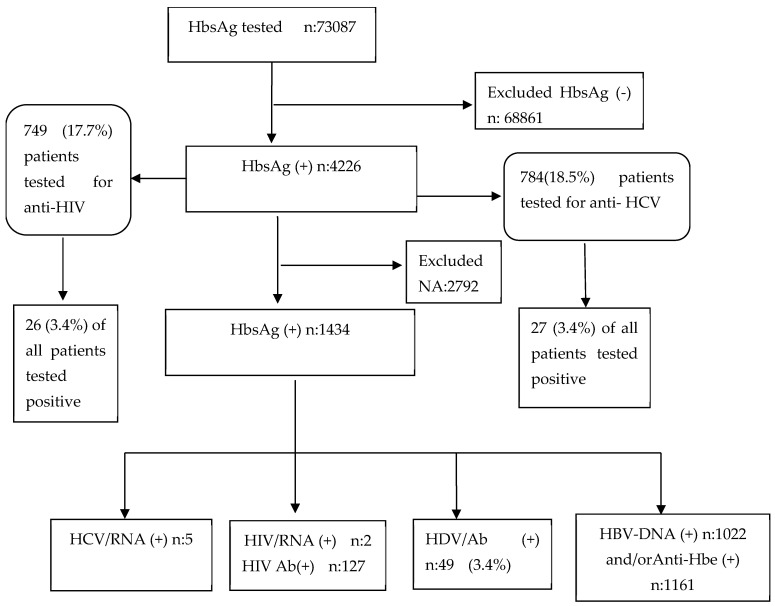
Flowchart of study selection.

A total of 73,087 samples were evaluated, and 68,861 HbsAg-negative samples were found. Among 4226 HbsAg-positive samples, 1434 patients whose data (hemogram, biochemistry, and HDV antibody) were obtained were included in the study. The rate of anti-HDV testing in HbsAg-positive individuals was found to be 33.9%. Among these patients, 49 (3.4%) had positive anti-HDV test results. Simultaneously, 784 patients were screened for anti-HCV, and 27 (3.4%) were found to be positive, whereas 749 patients were analysed for anti-HIV, and 26 (3.4%) were found to be positive (Figure 1).

The mean age of patients who tested positive for HBsAg was 48.3 years for the anti-HDV-negative group, and 43.18 years for the anti-HDV-positive group. Among the participants, 646 (45%) were female, and 788 (54.9%) were male. Anti-HCV tests were performed simultaneously in 784 patients (18.5%), and anti-HIV tests were performed in 749 patients (17.7%). The HCV antibody test yielded a positive result in 27 (3.4%) HbsAg-positive patients. Concurrently, the HIV antibody test yielded a positive result in 26 (3.4%) of the samples. The demographic data and serological and biochemical parameters, including alanine aminotransferase (ALT), aspartate transaminase (AST), bilirubin, albumin, international normalised ratio (INR), and platelet levels, as well as the mean APRI and FIB-4 values, are presented in Table 1. When the data were stratified by sex, no statistically significant differences were found in the anti-HDV positivity rates between males and females (*p* = 0.922). A statistically significant difference was observed between age groups in terms of past exposure to HDV (*p* = 0.01). Patients who were anti-HDV-negative exhibited slightly lower levels of ALT, platelets, and bilirubin, whilst those who were anti-HDV-positive demonstrated decreased levels of AST, INR, APRI, and albumin. Nevertheless, these disparities were not deemed to be statistically significant (*p* > 0.05). HBV DNA positivity rates were slightly higher in the anti-HDV-positive group (69.3%) than in the anti-HDV-negative group (63.6%), but this difference was not statistically significant (*p* = 0.453). Similarly, anti-HBe positivity rates were comparable between the two groups (80.8% vs. 83.6%, *p* = 0.714). However, HBeAg positivity rates were significantly greater in the anti-HDV-positive group (4.08% vs. 0.43%, *p* = 0.028), and HDV RNA positivity was significantly greater in the anti-HDV-positive group (4.08% vs. 0.07%, *p* = 0.003) (Table 1).

A number of low-level positive and statistically significant associations were identified. These included low-level positive and statistically significant associations between HIV RNA (r = 0.096), HDV RNA (r = 0.159), anti-HIV (r = 0.166), and anti-HDV positivity (*p* < 0.05). Furthermore, a significant correlation was identified between FIB-4 score and HDV antibody positivity (r = −0.057, *p* < 0.05).

**Figure 2:**
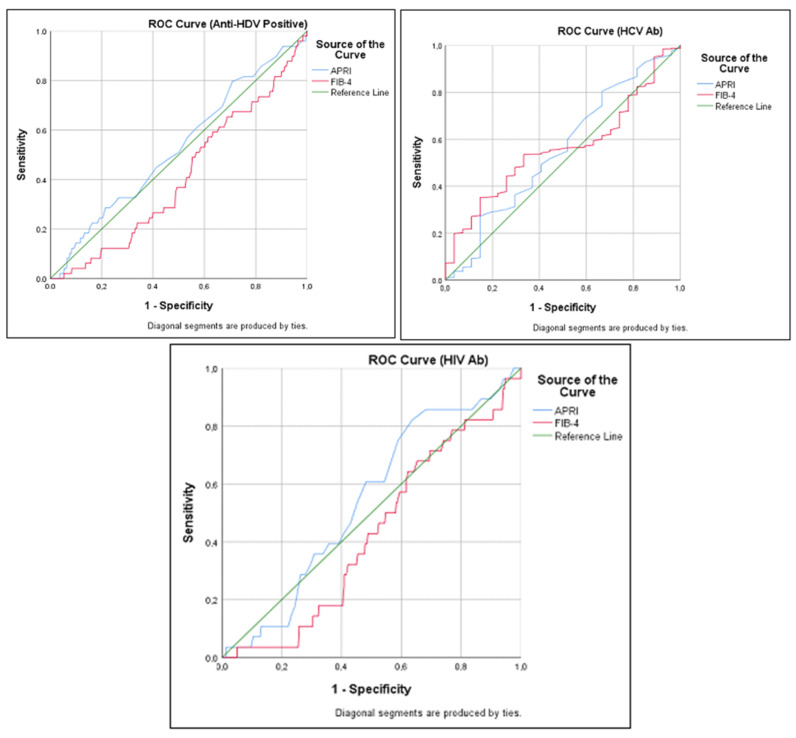
Estimation of the probability of anti-HDV, anti-HCV, and anti-HIV positivity using FIB-4 and APRI scores and ROC analysis.

According to ROC analysis, the FIB-4 score had a statistically significant effect on predicting anti-HDV negativity (AUC = 0.59, *p* = 0.031). However, the APRI score was not a significant predictor for anti-HDV positivity (AUC = 0.53, *p* > 0.05). APRI and FIB-4 scores did not have a statistically significant discriminatory power in predicting anti-HCV and anti-HIV positivity (*p* > 0.05). The FIB-4 score was the most significant predictor variable in terms of the cut-off value, sensitivity, and specificity. The cut-off value for the FIB-4 score in predicting anti-HDV positivity was 1.72, with a sensitivity of 61.4% and a specificity of 42.9% (*p* = 0.031) (Figure 2, Table 2).

The discriminatory capacity of APRI and FIB-4 scores in predicting exposure to HCV and HIV was found to be low, with no statistically significant results obtained (*p* > 0.05). The cut-off value of the FIB-4 score for exposure to HCV was determined as 1.83, with a sensitivity of 54.8% and a specificity of 55.6%. A similar outcome was observed when the FIB-4 score cut-off value for exposure to HIV was determined to be 1.88, with a sensitivity of 52.1% and a specificity of 57.1%.

The effects of APRI and FIB-4 scores on the probability of HDV Ab, HCV Ab, and HIV Ab positivity were analysed. The FIB-4 score was found to be a significant predictor for exposure to HDV (*p* = 0.016, OR = 0.655). However, no significant association was found for the APRI score (*p* > 0.05). APRI and FIB-4 scores were not found to be statistically significant predictors for exposure to HCV and HIV (*p* > 0.05). The odds ratio of the FIB-4 score for exposure to HCV was 0.715, and no significant effect was found (*p* > 0.05). For exposure to HIV, the odds ratio of FIB-4 score was 0.678, and no statistically significant relationship was observed (*p* > 0.05) (Table 3).

All five patients positive for HCV RNA were female. Among these patients, three were also positive for HBV-DNA, whereas the remaining two had positive anti-HBV results but undetectable HBV-DNA levels. Among the two patients who were positive for HIV-RNA, one was from a foreign nation with confirmed anti-HDV positivity, indicating HIV/HBV/HDV coinfection.

## 4. Discussion

In this study, the prevalence of anti-HDV, anti-HCV, and anti-HIV positivity among HBsAg-positive patients and the diagnostic performance of APRI and FIB-4 scores in predicting HDV, HIV, and HCV in HBsAg-positive patients were evaluated.

Research conducted within the borders of our nation has indicated that the prevalence of anti-HDV positivity ranges from 2% to 5%, exhibiting geographical variations and disparities among at-risk demographics [3,4,15]. In the present study, the anti-HDV positivity rate was 3.4%; furthermore, the rate of exposure to both HIV and HCV was 3.4 percent. The fact that 33.9% (n = 1434) of HbsAg-positive patients were screened for HDV antibodies indicates that the awareness level of responsible physicians should be increased. HbsAg-positive patients were screened for anti-HCV in 18.5% and for anti-HIV in 17.7% of individuals. In contrast, countries with higher HDV endemicity, such as parts of Africa and Eastern Europe, report prevalence rates as high as 20–30% among HBsAg-positive individuals [21,22]. Curici et al. reported coinfection rates of 11.3% for HDV, 1.4% for HCV, and 0.45% for HIV in their study evaluating the frequency of HDV, HCV, and HIV coinfection in patients with HBV [23]. These discrepancies highlight the regional diversity of HDV prevalence and the importance of local epidemiological data in guiding clinical strategies. Anti-HCV (3.4%) and anti-HIV (3.4%) positivity rates were detected in our study and are consistent with previous studies reporting regional seroprevalence differences. A nationwide Turkish study reported anti-HCV positivity rates of 1.5–2.5% and anti-HIV rates of 0.1–1%, reflecting the relatively low burden of these coinfections among HBsAg-positive individuals in the region [24,25]. In our study, the rate of anti-HIV positivity was higher in HBsAg-positive individuals than in the general population, and HBV infection is an indicator of HIV infection. Sincan prison is one of the largest prisons in Turkey, where our hospital provides services. Sincan prison has a capacity of 6830 detainees and convicts in an area of 2,500,000 m^2^ and thirteen penal execution institutions of L-type and F-type security levels. Globally, declining rates of HBV–HCV coinfections have been attributed to the widespread availability of direct-acting antiviral (DAA) therapies for HCV, which have drastically reduced the HCV burden in many countries [26,27]. A modelling study was conducted in our country to investigate the epidemiology and economic burden of HCV. The study concluded that the implementation of screening and treatment programmes for injectable drugs has the potential to reduce the number of deaths among prisoners and immigrants by almost two-thirds [28].

In the present study, the effects of APRI and FIB-4 scores on the probability of anti-HDV, anti-HCV, and anti-HIV positivity were examined. The FIB-4 score was found to be a significant predictor for anti-HDV positivity (*p* = 0.016, OR = 0.655). However, no significant association was found for the APRI score (*p* > 0.05). APRI and FIB-4 scores were not found to be statistically significant predictors for anti-HCV and anti-HIV positivity (*p* > 0.05). The odds ratio of the FIB-4 score for anti-HCV positivity was 0.715, and no significant effect was found (*p* > 0.05). For anti-HIV positivity, the odds ratio of the FIB-4 score was 0.678, and no statistically significant relationship was observed (*p* > 0.05). These findings suggest that the FIB-4 score may be an indicator in determining the probability of being anti-HDV-positive, but it is not an effective marker for anti-HCV and anti-HIV positivity. In a study by Gür-Altunay et al. at 2023, the gold standard method of biopsy was utilised to measure APRI and FIB-4 scores in 202 patients with chronic hepatitis B. The sensitivity and specificity of the APRI score were reported as 34% and 79%. The sensitivity and specificity of the FIB-4 score were reported as 71% and 46%, respectively. The study concluded that noninvasive tests can be employed to detect liver fibrosis in patients with chronic HBV [17]. Takyar et al. evaluated various serum fibrosis markers, including FIB-4 and the APRI, in patients with HDV infection in a study including 1003 patients with HCV (n = 701), HBV (n = 240), and HDV (n = 62). ROC analysis revealed scores of FIB-4 = 0.83 and APRI = 0.75 for HDV. The authors reported that noninvasive markers were limited in patients with chronic HDV infections and were better predictive markers of HCV and HBV monoinfection [18].

Abdel-Hameed et al. at 2021. evaluated the performance of the ELF index compared with the APRI and FIB4 in HIV/HCV coinfection in 147 HIV-/HCV-coinfected patients and 98 HCV-monoinfected patients. They reported that the APRI and FIB4 scores were poor predictors of liver fibrosis, regardless of HIV coinfection status [29]. A study conducted by Xu et al. investigated whether the FIB4 value could be used in screening for HBV, HCV, and nonalcoholic fatty liver disease (NAFLD). FIB-4 can reportedly be used as a screening tool for the secondary prevention of NAFLD in high-risk populations [30]. Jin et al. at 2012 analysed nine studies with a total of 1798 patients and investigated the diagnostic accuracy of the APRI and FIB4 values. They reported that the AUCs of the APRI for fibrosis and cirrhosis were 0.79 and 0.75, respectively. They reported that the APRI serum level is a limited indicator of HBV-related cirrhosis [31]. The findings of the present study indicated that the cut-off value of the FIB-4 score for anti-HCV positivity was determined as 1.83, with a sensitivity of 54.8% and a specificity of 55.6%. A similar result was observed when the cut-off value of the FIB-4 score for anti-HIV positivity was determined as 1.88, with 52.1% sensitivity and 57.1% specificity. The relatively small sample size of individuals who were HIV- and HCV-positive in the present study may have had an impact on the findings.

Despite the limited AUC values, the cut-off value for the FIB-4 score in predicting anti-HDV positivity was 1.72, with a sensitivity of 61.4% and a specificity of 42.9%. This result shows that the FIB-4 score shows significant discrimination in predicting the probability of being anti-HDV-negative. However, the low specificity value suggests that the power of the test to discriminate positive cases is limited. These findings suggest that the APRI and FIB-4 can serve as screening tools in resource-limited settings, where access to HDV/RNA testing or advanced imaging modalities (e.g., transient elastography) may be restricted. By facilitating early risk stratification, these noninvasive markers can optimise resource allocation and enable targeted follow-up for high-risk individuals. This approach aligns with global efforts to integrate cost-effective diagnostic algorithms into routine hepatitis management, particularly in underserved regions.

One major limitation of this study was the lack of reflex HDV/RNA testing for anti-HDV-positive patients. Reflex testing could increase the diagnostic accuracy for identifying active HDV infection, as anti-HDV positivity alone does not confirm active viremia.

The routine implementation of reflex HDV RNA testing in HBsAg-positive patients, particularly in regions with intermediate-to-high HDV endemicity, has been strongly recommended by recent guidelines [13,32,33]. Adopting such protocols could reduce underdiagnosis, improve clinical decision-making, and allow for the timely initiation of antiviral therapies tailored to HDV infection. Vaccination programmes are a key strategy to stop the spread of HDV [34].

The retrospective, single-centre design of this study is a limitation in terms of the generalisability of the findings, and the low prevalence of coinfections in this cohort constrained the statistical power for subgroup analyses. Future research should focus on multicentre studies involving larger patient cohorts to validate the results and explore novel biomarkers and imaging modalities for coinfection detection. The integration of sophisticated diagnostic techniques, such as FibroScan and proteomics-based fibrosis markers, into routine clinical practice has the potential to increase diagnostic accuracy and improve patient outcomes.

## 5. Conclusions

In conclusion, noninvasive scores (FIB-4 and APRI) do not show sufficient sensitivity and specificity in terms of HDV, HCV, and HIV exposure. Therefore, all HBsAg-positive patients should undergo routine anti-HDV testing, and vaccination programmes are vital in preventing the spread of HDV. Dual screening strategies are essential to identify infected individuals and develop prevention and treatment programmes. It is recommended that individuals who test positive for HBsAg should be screened for other common routes of transmission such as HIV and HCV as well as HDV, taking into account the use of noninvasive markers.

## Figures and Tables

**Table 1:** Demographic, serological, and biochemical characteristics of HBsAg-positive patients based on anti-HDV status.

Variables		Anti-HDV-Negative (n = 1385, 96.5%)		Anti-HDV-Positive (n = 49, 3.4%)		*p*
		n	%	Sayı	%	
Sex	Female	623	45.0	22	46.8	0.922
	Male	762	55.0	25	53.2	
		Ort. ± S.S. Med. (Min.–Max.)		Ort.±S.S. Med. (Min.–Max.)		
Age ^t^		48.3 ± 13.77 48 (18–95)		42.77 ± 12.17 42 (20–74)		0.007 **
HBsAg ^t^		3666.47 ± 1943.98 4487.33 (1.19–8278)		3736.56 ± 1672.73 4524.16 (8.51–5489.21)		0.807
HBV DNA ^z^		3.53 ± 1.58 3.41 (0.02–10.88)		3.96 ± 1.62 3.89 (1–9.84)		0.089
Anti-Hbe ^t^		24.02 ± 40.3 0.02 (0–100)		15.69 ± 34.56 0.01 (0–100)		0.144
Anti-HCV ^z^		0.12 ± 0.11 0.08 (0.01–0.93)		0.08 ± 0.07 0.06 (0.01–0.29)		0.064
Anti-HIV ^z^		0.1 ± 0.11 0.07 (0.01–1.07)		0.12 ± 0.11 0.1 (0.01–0.55)		0.045 *
ALT ^z^		29.23 ± 30.7 21 (4–339.2)		28.89 ± 24.18 23 (6–128.1)		0.823
AST ^z^		27.38 ± 25.53 21.4 (7–353)		25.82 ± 9.83 22.5 (13–59.2)		0.131
Albümin ^z^		4.22 ± 0.32 4.2 (2.8–5.4)		4.18 ± 0.29 4.2 (3.4–4.9)		0.103
Bilirubin ^z^		0.66 ± 0.34 0.56 (0.21–4.29)		0.68 ± 0.33 0.6 (0.27–1.68)		0.715
Platelet ^t^		251.35 ± 58.21 245 (154–589)		265.4 ± 66.91 255 (166–485)		0.162
INR ^z^		1.04 ± 0.11 1.02 (0.78–2.2)		1.01 ± 0.08 1 (0.7–1.2)		0.226
APRI ^z^		0.38 ± 0.4 0.3 (0.09–7.26)		0.35 ± 0.18 0.29 (0.09–0.97)		0.761
FIB-4 ^t^		2.15 ± 1.02 2 (0.26–6.46)		1.81 ± 0.82 1.79 (0.37–4.03)		0.022 *
HBV VL (logIU/mL)		3.53 ± 1.58		0.358 (0.08–0.967)		0.04
HbeAg positivity n (%)		6 (0.43)		2 (4.08)		0.028
Anti-Hbe positivity n (%)		1120 (80.8)		41 (83.6)		0.714
HBV DNA positivity n (%)		882 (63.6)		34 (69.3)		0.453

* *p* < 0.05; ** *p* < 0.01, t: independent sample *t*-test, z: Mann–Whitney U Test; categorical data: Chi-Square Test; VL: viral load.

**Table 2:** ROC analysis results for the prediction of anti-HDV, anti-HCV, and anti-HIV positivity probability.

Variables		Area *	Std. Error	*p*	Cut-Off Value	Sensitivity (%)	Specificity (%)	Confidence Interval (95)	
								Min	Max
HDV Ab (+)	APRI (a)	0.53	0.04	0.503	0.29	57.10	46.50	0.45	0.61
	FIB-4 (b)	0.59	0.04	0.031 *	1.72	61.40	42.90	0.52	0.67
HCV Ab (+)	APRI (a)	0.55	0.06	0.341	0.27	59.80	48.10	0.44	0.67
	FIB-4 (a)	0.56	0.05	0.277	1.83	54.80	55.60	0.47	0.65
HIV Ab (+)	APRI (a)	0.54	0.05	0.464	0.31	53.60	54.70	0.45	0.64
	FIB-4 (b)	0.58	0.05	0.176	1.88	52.10	57.10	0.48	0.67

* Area Under The Curve (AUC). Variables marked with the letter a were used to predict positivity, and FIB-4 scores and APRI scores marked with the letter b were used to predict negativity.

**Table 3:** Evaluation of variables affecting the probability of exposure to HDV, HCV, and HIV via Logistic Regression Analysis.

Dependent Variables	Predictor Variables	B	S.E.	*p*	Exp (B)/ Odds Ratio	95 C.I. for EXP (B)	
						Min	Max
HDV/Ab (+)	APRI	−0.44	0.53	0.406	0.644	0.23	1.82
	FIB-4	−0.42	0.18	0.016 *	0.655	0.47	0.92
HCV/Ab (+)	APRI	−0.16	0.61	0.795	0.854	0.26	2.80
	FIB-4	−0.34	0.23	0.14	0.715	0.46	1.12
HIV/Ab (+)	APRI	−0.21	0.60	0.722	0.807	0.25	2.63
	FIB-4	−0.39	0.23	0.091	0.678	0.43	1.06

* *p* < 0.05; Exp (B); odds ratio (OR).

## Data Availability

The data presented in this study are available on request from the corresponding author. The data are not publicly available due to privacy or ethical restrictions.
